# Prophylactic negative pressure wound therapy (NPWT) in laparotomy wounds (PROPEL-2): protocol for a randomized clinical trial

**DOI:** 10.1093/bjsopen/zrae081

**Published:** 2024-08-23

**Authors:** Matthew G Davey, Noel E Donlon, Stewart R Walsh, Claire L Donohoe, PROPEL-2 Trial Collaborators, C A Fleming, C Peirce, J C Coffey, E Condon, S A Elwahab, P W Owens, M E Kelly, J O Larkin, J B Conneely, M Varzgalis, M O'Riordain, E Faul, D P Toomey, D Winter, E Andrews, D E Kearney, P A Carroll, D Kavanagh, T Murphy, S T Martin, H M Heneghan, M K Barry, R A Cahill, P Neary, F Cooke, S T Johnston, W B Robb, A D K Hill, M J Kerin, J V Reynolds, D McNamara, S R Walsh

**Affiliations:** Department of Surgical Affairs, Royal College of Surgeons in Ireland, Dublin, Republic of Ireland; National Surgical Research Support Centre (NSRSC), Dublin, Republic of Ireland; Department of Surgical Affairs, Royal College of Surgeons in Ireland, Dublin, Republic of Ireland; National Surgical Research Support Centre (NSRSC), Dublin, Republic of Ireland; Department of Surgical Affairs, Royal College of Surgeons in Ireland, Dublin, Republic of Ireland; National Surgical Research Support Centre (NSRSC), Dublin, Republic of Ireland; Department of Surgical Affairs, Royal College of Surgeons in Ireland, Dublin, Republic of Ireland; National Surgical Research Support Centre (NSRSC), Dublin, Republic of Ireland

## Abstract

**Background:**

A proportion of patients undergoing midline laparotomy will develop surgical site infections after surgery. These complications place considerable financial burden on healthcare economies and have negative implications for patient health and quality of life. The prophylactic application of negative pressure wound therapy devices has been mooted as a pragmatic strategy to reduce surgical site infections. Nevertheless, further availability of multicentre randomized clinical trial data evaluating the prophylactic use of negative pressure wound therapy following midline laparotomy is warranted to definitely provide consensus in relation to these closure methods, while also deciphering potential differences among subgroups. The aim of this study is to determine whether prophylactic negative pressure wound therapy reduces postoperative wound complications in patients undergoing midline laparotomy.

**Methods:**

PROPEL-2 is a multicentre prospective randomized clinical trial designed to compare standard surgical dressings (control arm) with negative pressure wound therapy dressings (Prevena™ and PICO™ being the most commonly utilized). Patient recruitment will include adult patients aged 18 years or over, who are indicated to undergo emergency or elective laparotomy. To achieve 90% power at the 5% significance level, 1006 patients will be required in each arm, which when allowing for losses to follow-up, 10% will be added to each arm, leaving the total projected sample size to be 2013 patients, who will be recruited across a 36-month enrolment period.

**Conclusion:**

The PROPEL-2 trial will be the largest independent multicentre randomized clinical trial designed to assess the role of prophylactic negative pressure wound therapy in patients indicated to undergo midline laparotomy. The comparison of standard treatment to two commercially available negative pressure wound therapy devices will help provide consensus on the routine management of laparotomy wounds. Enrolment to PROPEL-2 began in June 2023.

**Registration number:** NCT05977816 (http://www.clinicaltrials.gov).

## Teaser

The PROPEL-2 prospective, randomized study looking to evaluate the role of negative pressure wound therapy for laparotomy wounds.

## Introduction

In recent decades, contemporary surgical practice has focused upon optimizing perioperative factors to enhance clinical outcomes for patients indicated to undergo surgery^1^. Surgical site complications remain a significant burden on surgical practice^2^, having negative implications on patient health, quality of life metrics and the cost effectiveness of the provision of surgical care. Surgical site infections (SSIs) are defined as incision or deep tissue infections at the operation site occurring up to 30 days after surgery^3^. SSIs constitute negative implications for surgical patients' postoperative recovery, through prolonging inpatient hospital stays, necessitating additional antimicrobial treatment, and in severe cases, leading to reintervention^4,5^.

Recent efforts have focused on identifying novel strategies to reduce SSIs^6,7^, yet the average incidence remains consistent in 0.5–3.0% of all surgical patients^8^ and 11.0% of general surgery patients^9^. Randomized clinical trial (RCT) data illustrates that SSIs have an incidence of 15–25% following colorectal and emergency abdominal surgery^10–12^.

The hypothesis supporting the use of suction-based drainage of infected wounds was first described by Soviet scientists in the mid-1980s for complex, contaminated wounds, with these preliminary results supporting their use in such cases^13,14^. The prophylactic application of negative pressure wound therapy (NPWT) devices was subsequently proposed as a novel strategy to combat chronic wound infections, following the publication of promising results from an animal study in 1997^15^. Thereafter, the application of NPWT for wound complications came into vogue^16,17^: in 2016, Hyldig *et al*. performed a meta-analysis of 10 RCTs which illustrated a relative risk reduction of 50% for wound infection and seroma formation relative to standard dressings^18^, albeit results were limited due to heterogeneity between the studies included. Sahebally *et al*. then performed a meta-analysis of 1266 patients which demonstrated a 75% relative reduction in SSI for patients who had NPWT applied to their closed abdominal surgery wounds^19^.

PICO™ (Smith & Nephew, London, UK) and Prevena™ (KCI, 3M, St Paul, MN, USA) wound management systems are among the most widely used NPWT in surgical practice. Importantly, the PICO trial performed in 2017 illustrated a significant reduction in SSI rate and duration of hospital stay when compared with standard wound dressings^20^. Conversely, the NEPTUNE trial failed to identify any difference in wound infection rates postlaparotomy when comparing Prevena™ to standard dressings in patients undergoing colorectal surgery^21^. Data from the CHIR-Net, PROUD and ROSSINI RCTs demonstrated an incidence rate of 15–25% following gastrointestinal surgery^10–12^, with significant morbidity rates and costs^22^. Furthermore, most trials included short durations of follow-up (median: 7 days)^23^, with no trials comparing different types of NPWT and conflicting results from different devices trialled^21,22^. Additionally, there are limited data available on economic analyses for gastrointestinal surgery indications^23^. Accordingly, the necessity to identify novel interventions to reduce SSI incidence is imperative, yet given the heterogeneity of results, there are no consensus statements or guidelines recommending the routine use of prophylactic NPWT to prevent SSI postlaparotomy.

Thus, the aim of the PROPEL-2 trial is to determine whether NPWT confers a lower rate of SSI following laparotomy. The hypothesis is that patients randomized to NPWT will have a lower incidence of SSI.

## Methods

### Trial design

PROPEL-2 is a prospective, multicentre RCT recruiting patients from surgical units across the Republic of Ireland designed in accordance with the CONSORT guidelines for prospective, two parallel group randomized studies^24^. This trial was prospectively registered at http://www.clinicaltrials.gov as NCT05977816.

Patients recruited to this study may be randomized to Standard dressing or NPWT (that is: PICO™, Prevena™, etc.). The control arm will be randomized to receive a standard transparent waterproof dressing.

PROPEL-2 will have two phases. Phase I (internal pilot) is designed to confirm the expected recruitment rate for a large-scale multicentre RCT. Phase 2 (main phase) will be the proposed RCT in centres across the island of Ireland.

#### Internal pilot summary

The pilot will be conducted over an interval of 6 months, including secondary and tertiary referral centres. The internal pilot aims to determine the number of eligible and recruited patients in the centres over the course of 6 months. If recruitment targets are not achieved a review of the reasons will be investigated in terms of ineligibility due to protocol eligibility requirements, poor site performance and lack of suitable patient population. If targets are met, and any issues resolved, the trial will proceed into the main phase, including patients from the internal pilot. If the trial does not progress to Phase 2, those participants already enrolled in Phase 1 will be followed up as per protocol.

All adult patients presenting at trial centres requiring surgery via a midline laparotomy incision as the primary operative access are eligible for inclusion. Patients will be excluded if pregnant, if they have undergone a relook laparotomy, if their abdomen is left open or if unable to adhere. Randomization will be stratified by trial centre, emergency or elective procedure, and surgical specialty (upper gastrointestinal, lower gastrointestinal, general, vascular, urology). Randomization will be generated and administered via a secure web service, Sealed Envelope, using minimization. The random allocation will be to either standard wound dressing or single-use NPWT. Participating patients will have clinical follow-up for 6 months. Wound photographs will be taken at 1-month, 3-month and 6-month visits. The photograph at 1 month will assist with diagnosis of any infection. Photographs from the 1-month, 3-month and 6-month visits will be used by the clinician to evaluate wound healing together with a patient-reported assessment using the patient and observer scar assessment scale (POSAS) questionnaire^25^. EuroQol 5-Dimension 5-level (EQ5D-5L) quality of life questionnaires will be completed before surgery and at 1 month, 3 months and 6 months to collect quality of life outcome data^26^. At 3 and 6 months, the patient will complete a Cost for Patients Questionnaire (CoPaQ) to evaluate the financial impact on the patient^27^. Information regarding resource use, late complications or additional interventions related to their index procedure will be used to evaluate the financial impact on the healthcare system. Patient monitoring will be performed by the clinical teams responsible for the management of these patients after surgery.

### Treatment arms

#### Standard dressing

The standard dressing for a laparotomy wound comprises a non-adhesive layer applied directly to the wound covered by a sealed dressing and does not use negative pressure.

The materials used will be at the discretion of the surgeon but details of each NPWT dressing applied will be noted. Acceptable standard dressings include Mepore© (Molnlycke Healtcare, Gothenberg, Sweden), Opsite© (Smith and Nephew, London, UK) and Tegadrem© (3M, MN, USA), among others.

### Negative pressure wound therapy

NPWT dressings use an open cell foam laid on top of the wound. It is then covered with an impermeable adhesive membrane. A sealed tube punctures the membrane to connect the dressing to the in-built pump leading to the creation of a partial vacuum of the wound. Generally, the first dressing applied at the end of the operation is left in place until the wound is ready for sutures to be removed at approximately 7–10 days. The number of replacement NPWT dressings within the first 30 and 90 days will be captured.

The Prevena^TM^ (Kinetic Concepts Inc, San Antonio, TX, USA) Incision Management system is comprised of a suction unit that has a small canister for fluid collection, tubing and a sponge dressing with a non-adherent layer and semipermeable adhesive drape. Prevena^TM^ contains a 0.019% ionic silver solution which has utility in preventing bacterial growth in the dressing. The system is ergonomic as it is supplied with a carrying case to minimize negative implications on patient mobility. When applied and activated, this system maintains a closed environment and removes any potential fluid which may collect at the wound site via the application of negative pressure therapy.

The PICO^TM^ (Nephew Wound Management, London, UK) is a NPWT system that consists of a silicone wound interface dressing that is applied directly onto the wound. The purpose of this interface is to transmit even pressure distribution across the wound bed, while negative pressure is transmitted from the wound filler at −80 mmHg.

### Study objectives

The primary objective for the trial is to quantify and analyse differences in the rates of midline wound SSI in the 30 and 90 days following laparotomy between NPWT and standard dressings. The diagnosis of SSI will be made using Centre for Disease Control criteria.

The secondary objectives are to analyse health-related quality of life in the 6 months following laparotomy and to assess the quality of wound healing and cost effectiveness. Another objective is to analyse the further interventions related to laparotomy and the risk of developing SSI related to the subcutaneous adiposity and lean muscle mass using preoperative computed tomography (CT). In a nested Study Within A Trial (SWAT), another objective is to determine the impact of digital prompts (for example using e-mail) to research team members on site trial recruitment rates.

### Outcome measures

#### Primary outcome measure

The primary outcome measure is the incidence of any SSI. The treating clinical team who will make the diagnosis will not be members of the research team. For the purposes of the trial, any wound infection requiring ongoing medical intervention/which has led to reoperation at or after the 30 and 90 day review will be considered a deep SSI.

#### Secondary outcome measures

**Fig. 1.**
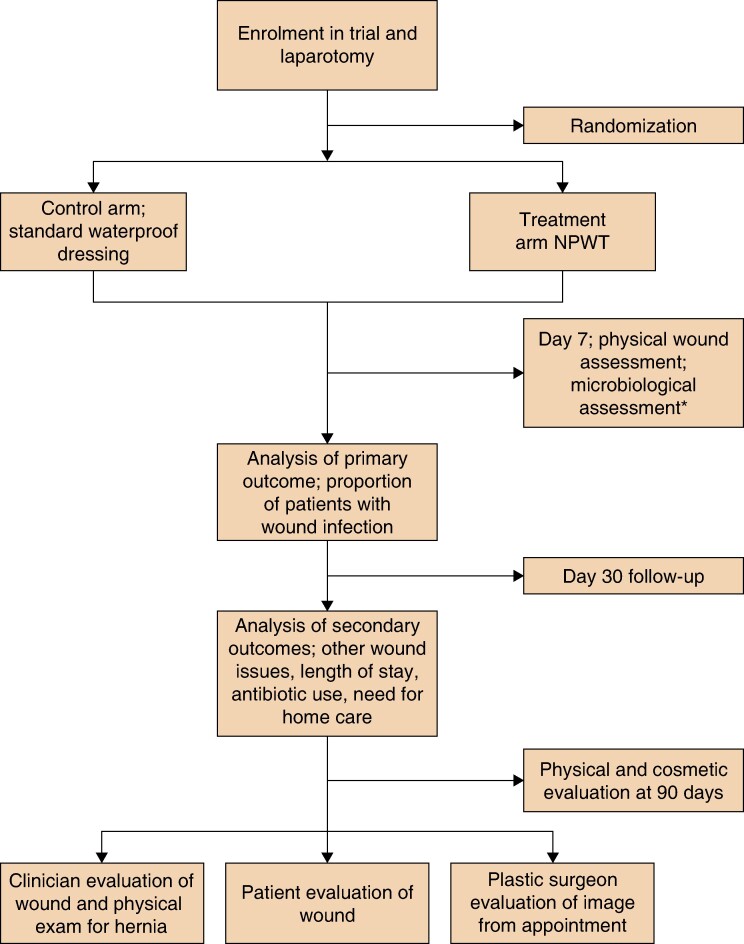
CONSORT diagram for the PROPEL-2 trial

The secondary outcome measures in this trial are to compare the use of standard dressings with NPWT dressings in terms of impact on quality of life (QoL), with the EuroQoL EQ-5D-5L questionnaire and Quality Adjusted Life Year (QALY) profiles^26^, wound healing and scar appearance (using the POSAS questionnaire and the photograph taken at each follow-up visit)^25^, postoperative complications (obtained from the patient chart for this study) and economic settings (using the validated CoPaQ questionnaire for patients^27^). Incidence rates of skin blistering, allergic reactions to dressings and device/dressing malfunction were also evaluated. *Figure 1* outlines the CONSORT diagram for the PROPEL-2 trial.

*To be performed by teams responsible for patient care. NPWT, negative pressure wound therapy.

A subanalysis evaluating the relationship between preoperative subcutaneous adiposity and lean muscle mass on postoperative SSI will be performed. Adiposity and muscle mass data will be collected using preoperative computed tomography images. A SWAT will assess the impact of digital prompts on trial recruitment rates.

### Power calculation

For planning purposes, it is assumed 15% of participants randomized to standard dressings will develop an SSI and the rate among patients receiving prophylactic, single-use NPWT will be 10% (that is a relative risk reduction of 33%, absolute risk reduction 5%). To achieve 90% power at the 5% significance level, a minimum of 1006 patients will be required in each arm. To allow for losses to follow-up and withdrawals, 10% will be added to each arm. Therefore, the total projected sample size for PROPEL-2 is 2013 patients. This power calculation for PROPEL-2 did not estimate or account for subgroups, including elective and emergency laparotomy wounds or surgical procedures performed.

### Ethical approval

The PROPEL-2 trial is designed in accordance with the principles of the Declaration of Helsinki and ‘good clinical practice’ guidelines^28^. The protocol has been approved by the ethics committee at Tallaght University Hospital/St. James Hospital, Dublin, and approval has been obtained from other participating centres. Written informed consent will be prospectively obtained before surgery. This study will receive no financial aid.

### Randomization

Eligibility will be confirmed by the treating surgeon at the end of the operative procedure before the wound dressing is applied. Randomization will be on a one-to-one basis using a computer randomization programme with the minimization algorithm to ensure a balanced allocation of patients across the two treatment groups, stratified by trial centre, emergency or elective procedure, and surgical specialty. The first 30 participants will be randomized using simple randomization in order to seed the minimization algorithm, which will have a probable cystic element incorporated to ensure unpredictability of treatment assignment.

### Wound closure

Wound closure will be performed in a standardized fashion, as outlined in detail below. Each recruitment centre will use a wound protector upon entering the peritoneal cavity and at all times attempt to keep contamination to a minimum. Surgical gloves will be changed before commencement of closure and a new instrument set will be used. The wound edges will be washed with povidone iodine (Bethidine©, Mundipharma, Cambridge, UK) following removal of the wound protector.The fascial layer will be closed with slowly absorbable monofilament (that is: 1–0 loop polyester poly(p-dioxanone) or PDS) (Ethicon Inc., Rariton, NJ, USA). The skin will then be closed with skin clips with the wounds appropriately everted. The depth of the subcutaneous tissue will be recorded with a sterile ruler from a pen kit. The length of the laparotomy wound will be recorded and then the randomized dressing will be applied.

If the NPWT fails or if an SSI is recorded, a new NPWT dressing will be applied. For the standard dressing group, a new standard dressing will be applied. In such cases, antibiotics will be empirically started and decisions regarding further surgical intervention will be made at the discretion of the consultant surgeon.

### Patient trial identification number assignment

The patient trial number will then be used to enter patient data on a secure, encrypted, web-based system, REDCap (ProjectREDCap, Nashville, TN, USA). Basic information regarding age and eligibility checks will be entered.

### Statistical analysis

Standard statistical summaries and graphical plots will be presented for the primary outcome measure and all secondary outcome measures. Baseline data will be summarized to check comparability between treatment arms.

The main analysis will investigate differences in the primary outcome measure: the proportion of patients with any SSI at 30 and 90 days postlaparotomy. While clustering effects are not expected to be important for this study, in reality the data will be hierarchical, with patients naturally clustered into groups by recruiting centre. This will be accounted for by generalizing the conventional linear (fixed-effects) regression approach to a mixed-effects logistic regression analysis. This model will be used to assess differences in SSI rates between the study intervention groups, with results presented as odds ratios with associated 95% confidence intervals. The mixed-effects model will include a random effect to account for any heterogeneity in response due to the recruitment centre and fixed effects to adjust for emergency *versus* elective procedure and surgical specialty. An identically structured and formulated mixed-effects linear regression model will be used to assess the effects of the interventions on secondary outcomes and EQ-5D and WoundQoL (at both 3 and 6 months) that, for the purposes of analysis, will be assumed to be approximately normally distributed. Other dichotomous outcome variables, such as complications related to the trial interventions, will be analysed in the same manner as the primary outcome. Temporal patterns of any complications will be presented graphically and if appropriate a time-to-event analysis (Kaplan–Meier survival analysis) will be used to assess the overall risk and risk within individual classes of complications.

The primary focus will be to compare the two treatment groups on an intention-to-treat (ITT) basis, and this will be reflected in the analysis which will be reported together with appropriate diagnostic plots that check the underlying model assumptions. In addition to the ITT analyses, per-protocol (as treated) analyses will also be undertaken and reported in parallel to, but subsidiary to, the main analyses. Where possible the reasons for missing data will be determined. Although missing data is not expected to be a problem for this study, the nature and pattern of the ‘missingness’ will be considered—including in particular, whether data can be treated as missing at random (MAR). If judged appropriate, missing data will be imputed, using multiple imputation. The resulting imputed data sets will be analysed and reported, together with appropriate sensitivity analyses. Any imputation methods used for scores and other derived variables will be reviewed by the Data Monitoring Committee (DMC). Reasons for ineligibility, non-compliance, withdrawal or other protocol violations will be stated if available and any patterns summarized. More formal analysis, for example using logistic regression with ‘protocol violation’ as a response, may also be appropriate and aid interpretation.

All reported tests will be two-sided and considered to provide evidence for a significant difference if *P* values are less than 0.05 (5% significance level). A detailed statistical analysis plan (SAP) will be agreed with the DMC before commencement of the study. Any subsequent amendments to this initial SAP will be clearly stated and justified in the final report. Interim analyses of efficacy outcomes are not planned and will be performed only where requested by the DMC. Data will be analysed using Statistical Package for Social Sciences™ (SPSS™) version 26.0 (International Business Machines Corporation, Armonk, NY, USA).

### Trial oversight

Day-to-day patient management will rest with the clinical teams responsible for the management of these patients after surgery, while day-to-day trial management will rest with the PROPEL Trial Group in the Irish Surgical Research Collaborative, who will have full responsibility for data collated and logged to the electrical database. Administrative support will be provided by the Royal College of Surgeons in Ireland (RCSI) National Surgical Research Support Centre (NSRSC). This will be overseen by the Trial Management Group (TMG), which will meet on a monthly basis to assess progress. A Trial Steering Committee (TSC) and DMC will be established.

### Trial supervision

The TMG will comprise investigators together with RCSI NSRSC staff involved in the trial. An independently chaired TSC and DMC will be convened. The TSC will provide overall supervision of the trial and will meet annually during trial recruitment. The roles of the TSC are to monitor and supervise the progress of the trial towards its objectives, review at regular intervals relevant information from other sources and consider the recommendations of the DMC.

The DMC is a group of independent experts external to the trial who assess the progress, conduct, participant safety and, if required, critical endpoints of a clinical trial. The study DMC will adopt a DAMOCLES charter which defines its terms of reference and operation in relation to oversight of the trial. They will not be asked to perform any formal analyses of effectiveness. They will review accruing data, provide summaries of the data presented by treatment group, and will assess the screening algorithm against the eligibility criteria. They will also consider emerging evidence from other related trials or research.

### Quality control

The NSRSC will provide an oversight of trial processes at each site. Quality assurance checks will be undertaken by the NSRSC to ensure integrity of randomization, study entry procedures and data collection. Furthermore the processes of consent taking, randomization, registration, provision of information and provision of treatment will be monitored. Written reports will be produced for the TSC, informing them of protocol deviations and if any corrective and prevention actions (CAPAs) are required.

The NSRSC or designee will conduct site monitoring in accordance with the study monitoring plan. This site monitoring will be an ongoing activity from the time of initiation until study close-out and will comply with the International Council for Harmonisation of Technical Requirements for Pharmaceuticals for Human Use Guideline for Good Clinical Practice (ICH-GCP). During site visits, the monitor will review original patient records, Clinical Research Facility (CRF) entries and essential documents. The monitor will discuss any problems with the investigator and CAPAs will be implemented if required. All remote and site monitoring visits will be documented in a monitoring visit report and the reports will be reviewed by the RCSI Sponsor Office and filed.

## Discussion

PROPEL-2 will be the largest standard parallel RCT designed to evaluate NPWT devices and standard dressings in reducing SSI. In surgical practice, using NPWT for complicated abdominal wounds is abundant^29^, yet there are few high-quality clinical trials supporting their prophylactic application following laparotomy. Sahebally *et al*. performed a meta-analysis of 1266 patients from eight studies (that is: three RCTs, two prospective and four retrospective cohort studies) which demonstrated a significant reduction in SSI rates following NPWT for closed abdominal surgery wounds^19^, which was later validated in the work of Boland *et al.*^30^. The PICO trial was among these studies, which demonstrated an absolute risk of SSI of 8.3% in the PICO™ group *versus* a 32.0% risk in those randomized to standard care dressings^20^, which translated to a significant reduction in hospital stay (6.1 days *versus* 14.7 days). The results of the PICO trial were subject to detection bias as the study assessor was not blinded to the treatment group when evaluating their results, suggesting that further validation of their findings in the multicentre setting is imperative before being implemented in the ‘standard’ following laparotomy, particularly when the subsequent NEPTUNE trial has refuted such findings^21^.

Suggestions have mounted surrounding the burden SSIs pose to healthcare services, with estimated costs of 90 million pounds per annum in the UK^31^. While the direct management of SSIs is inexpensive, the indirect costs associated with the increased duration of hospital stay, readmissions and prolonged follow-up render SSIs more expensive^32–34^. NPWT devices are disposable after single use and compact to facilitate mobilization^32^, characteristics which enable wound management in an outpatient setting. There is concern related to the considerable cost of NPWT^34^; this cost would be justified if it is shown that NPWT is able to reduce SSIs by 15%^20,32^.

The hypothesis supporting the use of NPWT to enhanced wound healing is driven by several fundamental concepts^35,36^. The hypoxic environment created through the sealed dressing reduces the bacterial bioburden in local tissues, thus facilitating improved wound healing^37–39^. This hypoxic environment facilitates increased levels of circulating interleukins and increased growth factor expression, which increase angiogenesis, extracellular matrix remodelling and granulation tissue formation^35^. Using NPWT is associated with increased stabilization and maturation of microvasculature in local tissues, increasing blood flow (particularly in the latter stages of wound healing)^40^.

Global surgery initiatives aim to support equity in healthcare to all patients worldwide^41,42^. SSI rates are significantly higher in the developing world^43^, thus the application of single-use NPWT systems as prophylaxis is easily transferable to such settings, and may prove cost effective for such healthcare economies. Furthermore, as novel modifications refine contemporary devices, their costs may decrease significantly, making them more cost effective and accessible to patients globally, irrespective of where they receive their medical care.

This study will be subject to certain unavoidable limitations. As with all RCTs in surgery, the apparent inability to blind surgeons and other stakeholders in patient care to the intervention inevitably lends such studies to be classified as ‘open label’, rendering them subject to potential biases. Furthermore, the failure to standardize the control arm of this study may render results subject to confounding biases.

## Collaborators

C.A. Fleming; C. Peirce; J.C. Coffey; E. Condon; S.A. Elwahab; P.W. Owens; M.E. Kelly; J.O. Larkin; J.B. Conneely; M. Varzgalis; M. O'Riordain; E. Faul; D.P. Toomey; D. Winter; E. Andrews; D.E. Kearney; P.A. Carroll; D. Kavanagh; T. Murphy; S.T. Martin; H.M. Heneghan; M.K. Barry; R.A. Cahill; P. Neary; F. Cooke; S.T. Johnston; W.B. Robb; A.D.K. Hill; M.J. Kerin; J.V. Reynolds; D. McNamara; S.R. Walsh.

## Data Availability

Data will be made available upon reasonable request from the PROPEL Trial Group in the Irish Surgical Research Collaborative.

## References

[1] Ljungqvist O , Scott M, Fearon KC. Enhanced recovery after surgery: a review. JAMA Surg 2017;152:292–298 28097305 10.1001/jamasurg.2016.4952

[2] Pearson A . Historical and changing epidemiology of healthcare-associated infections. J Hosp Infect 2009;73:296–304 19925942 10.1016/j.jhin.2009.08.016

[3] Owens CD , Stoessel K. Surgical site infections: epidemiology, microbiology and prevention. J Hosp Infect 2008;70:3–10 19022115 10.1016/S0195-6701(08)60017-1

[4] Smith RL , Bohl JK, McElearney ST, Friel CM, Barclay MM, Sawyer RG et al Wound infection after elective colorectal resection. Ann Surg 2004;239:599–605; discussion 605–607 15082963 10.1097/01.sla.0000124292.21605.99 PMC1356267

[5] Mangram AJ , Horan TC, Pearson ML, Silver LC, Jarvis WR. Guideline for Prevention of Surgical Site Infection, 1999. Centers for Disease Control and Prevention (CDC) Hospital Infection Control Practices Advisory Committee. Am J Infect Control 1999;27:97–132; quiz 133–134; discussion 96 10196487

[6] Ademuyiwa AO , Hardy P, Runigamugabo E, Sodonougbo P, Behanzin H, Kangni S et al Reducing surgical site infections in low-income and middle-income countries (FALCON): a pragmatic, multicentre, stratified, randomised controlled trial. Lancet 2021;398:1687–1699 34710362 10.1016/S0140-6736(21)01548-8 PMC8586736

[7] De Simone B , Sartelli M, Coccolini F, Ball CG, Brambillasca P, Chiarugi M et al Intraoperative surgical site infection control and prevention: a position paper and future addendum to WSES intra-abdominal infections guidelines. World J Emerg Surg 2020;15:10 32041636 10.1186/s13017-020-0288-4 PMC7158095

[8] Seidelman JL , Mantyh CR, Anderson DJ. Surgical site infection prevention: a review. JAMA 2023;329:244–252 36648463 10.1001/jama.2022.24075

[9] Gillespie BM , Harbeck E, Rattray M, Liang R, Walker R, Latimer S et al Worldwide incidence of surgical site infections in general surgical patients: a systematic review and meta-analysis of 488,594 patients. Int J Surg 2021;95:106136 34655800 10.1016/j.ijsu.2021.106136

[10] Diener MK , Knebel P, Kieser M, Schüler P, Schiergens TS, Atanassov V et al Effectiveness of triclosan-coated PDS plus versus uncoated PDS II sutures for prevention of surgical site infection after abdominal wall closure: the randomised controlled PROUD trial. Lancet 2014;384:142–152 24718270 10.1016/S0140-6736(14)60238-5

[11] Pinkney TD , Calvert M, Bartlett DC, Gheorghe A, Redman V, Dowswell G et al Impact of wound edge protection devices on surgical site infection after laparotomy: multicentre randomised controlled trial (ROSSINI trial). BMJ 2013;347:f4305 23903454 10.1136/bmj.f4305 PMC3805488

[12] Mihaljevic AL , Schirren R, Özer M, Ottl S, Grün S, Michalski CW et al Multicenter double-blinded randomized controlled trial of standard abdominal wound edge protection with surgical dressings versus coverage with a sterile circular polyethylene drape for prevention of surgical site infections: a CHIR-net trial (BaFO; NCT01181206). Ann Surg 2014;260:730–737; discussion 737–739 25379844 10.1097/SLA.0000000000000954

[13] Iu AD , Malafeeva EV, Smirnov AP, Flegontov VB. Vacuum therapy in the treatment of suppurative lactation mastitis. Vestn Khir Im I I Grek 1986;137:66–70 3824787

[14] Kostiuchenok BMK , Karlov VA II, Ignatenko SN, Muzykant LI. Vacuum treatment in the surgical management of suppurative wounds. Vestn Khir Im I I Grek 1986;137:18–21 3787969

[15] Morykwas MJ , Argenta LC. Nonsurgical modalities to enhance healing and care of soft tissue wounds. J South Orthop Assoc 1997;6:279–288 9434249

[16] Lord AC , Hompes R, Venkatasubramaniam A, Arnold S. Successful management of abdominal wound dehiscence using a vacuum assisted closure system combined with mesh-mediated medial traction. Ann R Coll Surg Engl 2015;97:e3–e5 25519257 10.1308/003588414X14055925059237 PMC4473924

[17] Mukhi AN , Minor S. Management of the open abdomen using combination therapy with ABRA and ABThera systems. Can J Surg 2014;57:314–319 25265104 10.1503/cjs.026613 PMC4183677

[18] Hyldig N , Birke-Sorensen H, Kruse M, Vinter C, Joergensen JS, Sorensen JA et al Meta-analysis of negative-pressure wound therapy for closed surgical incisions. Br J Surg 2016;103:477–486 26994715 10.1002/bjs.10084 PMC5069647

[19] Sahebally SM , McKevitt K, Stephens I, Fitzpatrick F, Deasy J, Burke JP et al Negative pressure wound therapy for closed laparotomy incisions in general and colorectal surgery: a systematic review and meta-analysis. JAMA Surg 2018;153:e183467 30267040 10.1001/jamasurg.2018.3467 PMC6583074

[20] O’Leary DP , Peirce C, Anglim B, Burton M, Concannon E, Carter M et al Prophylactic negative pressure dressing use in closed laparotomy wounds following abdominal operations: a randomized, controlled, open-label trial: the P.I.C.O. trial. Ann Surg 2017;265:1082–1086 27926575 10.1097/SLA.0000000000002098

[21] Murphy PB , Knowles S, Chadi SA, Vogt K, Brackstone M, Koughnett JAV et al Negative pressure wound therapy use to decrease surgical nosocomial events in colorectal resections (NEPTUNE): a randomized controlled trial. Ann Surg 2019;270:38–42 30499799 10.1097/SLA.0000000000003111

[22] Monahan M , Jowett S, Pinkney T, Brocklehurst P, Morton DG, Abdali Z et al Surgical site infection and costs in low- and middle-income countries: a systematic review of the economic burden. PLoS One 2020;15:e0232960 32497086 10.1371/journal.pone.0232960 PMC7272045

[23] Norman G , Goh EL, Dumville JC, Shi C, Liu Z, Chiverton L et al Negative pressure wound therapy for surgical wounds healing by primary closure. Cochrane Database Syst Rev 2020;5:CD009261 32356396 10.1002/14651858.CD009261.pub5 PMC7192856

[24] Moher D , Hopewell S, Schulz KF, Montori V, Gøtzsche PC, Devereaux PJ et al CONSORT 2010 explanation and elaboration: updated guidelines for reporting parallel group randomised trials. BMJ 2010;340:c869 20332511 10.1136/bmj.c869 PMC2844943

[25] Draaijers LJ , Tempelman FR, Botman YA, Tuinebreijer WE, Middelkoop E, Kreis RW et al The patient and observer scar assessment scale: a reliable and feasible tool for scar evaluation. Plast Reconstr Surg 2004;113:1960–1965; discussion 1966–1967 15253184 10.1097/01.prs.0000122207.28773.56

[26] Rabin R , de Charro F. EQ-5D: a measure of health status from the EuroQol group. Ann Med 2001;33:337–343 11491192 10.3109/07853890109002087

[27] Laberge M , Coulibaly LP, Berthelot S, da Silva B, Guertin JR, Strumpf E et al Development and validation of an instrument to measure health-related out-of-pocket costs: the cost for patients questionnaire. Value Health 2021;24:1172–1181 34372983 10.1016/j.jval.2021.03.016

[28] Devine S , Dagher RN, Weiss KD, Santana VM. Good clinical practice and the conduct of clinical studies in pediatric oncology. Pediatr Clin North Am 2008;55:187–209, xi–xii 18242321 10.1016/j.pcl.2007.10.008 PMC2276977

[29] Shweiki E , Gallagher KE. Negative pressure wound therapy in acute, contaminated wounds: documenting its safety and efficacy to support current global practice. Int Wound J 2013;10:13–43 22420782 10.1111/j.1742-481X.2012.00940.x PMC7950513

[30] Boland PA , Kelly ME, Donlon NE, Bolger JC, Mehigan BJ, McCormick PH et al Prophylactic negative pressure wound therapy for closed laparotomy wounds: a systematic review and meta-analysis of randomised controlled trials. Ir J Med Sci 2021;190:261–267 32588378 10.1007/s11845-020-02283-7 PMC7315908

[31] Payne C , Edwards D. Application of the single use negative pressure wound therapy device (PICO) on a heterogeneous group of surgical and traumatic wounds. Eplasty 2014;14:e20 24917894 PMC4006427

[32] Ramadhar AJ , Abraham F, McAllen C. “Gravity”—a new simple negative pressure wound therapy self-build design for low income countries. J Med Eng Technol 2018;42:518–524 30875268 10.1080/03091902.2019.1576791

[33] Heard C , Chaboyer W, Anderson V, Gillespie BM, Whitty JA. Cost-effectiveness analysis alongside a pilot study of prophylactic negative pressure wound therapy. J Tissue Viability 2017;26:79–84 27320010 10.1016/j.jtv.2016.06.001

[34] Urban JA . Cost analysis of surgical site infections. Surg Infect (Larchmt) 2006;7:S19–S22 16834543 10.1089/sur.2006.7.s1-19

[35] Glass GE , Murphy GF, Esmaeili A, Lai LM, Nanchahal J. Systematic review of molecular mechanism of action of negative-pressure wound therapy. Br J Surg 2014;101:1627–1636 25294112 10.1002/bjs.9636

[36] Huang C , Leavitt T, Bayer LR, Orgill DP. Effect of negative pressure wound therapy on wound healing. Curr Probl Surg 2014;51:301–331 24935079 10.1067/j.cpsurg.2014.04.001

[37] Weed T , Ratliff C, Drake DB. Quantifying bacterial bioburden during negative pressure wound therapy: does the wound VAC enhance bacterial clearance? Ann Plast Surg 2004;52:276–279; discussion 279–280 15156981 10.1097/01.sap.0000111861.75927.4d

[38] Banwell P , Withey S, Holten I. The use of negative pressure to promote healing. Br J Plast Surg 1998;51:79 10.1016/s0007-1226(98)80142-2 9577325

[39] López-Cano M , Armengol-Carrasco M. Use of vacuum-assisted closure in open incisional hernia repair: a novel approach to prevent seroma formation. Hernia 2013;17:129–131 21667262 10.1007/s10029-011-0837-6

[40] Ma Z , Shou K, Li Z, Jian C, Qi B, Yu A. Negative pressure wound therapy promotes vessel destabilization and maturation at various stages of wound healing and thus influences wound prognosis. Exp Ther Med 2016;11:1307–1317 27073441 10.3892/etm.2016.3083 PMC4812564

[41] Roa L , Jumbam DT, Makasa E, Meara JG. Global surgery and the sustainable development goals. Br J Surg 2019;106:e44–e52 30620060 10.1002/bjs.11044

[42] Quene TM , Bust L, Louw J, Mwandri M, Chu KM. Global surgery is an essential component of global health. Surgeon 2022;20:9–15 34922839 10.1016/j.surge.2021.10.001 PMC8695837

[43] Sawyer RG , Evans HL. Surgical site infection–the next frontier in global surgery. Lancet Infect Dis 2018;18:477–478 29452939 10.1016/S1473-3099(18)30118-X

